# The Effects of Mind-Body Exercise on Cognitive Performance in Elderly: A Systematic Review and Meta-Analysis

**DOI:** 10.3390/ijerph15122791

**Published:** 2018-12-09

**Authors:** Yanjie Zhang, Chunxiao Li, Liye Zou, Xiaolei Liu, Wook Song

**Affiliations:** 1 Health and Exercise Science Laboratory, Institute of Sports Science, Seoul National University, Seoul 08826, Korea; elite_zhangyj@163.com; 2 Physical Education Unit, School of Humanities and Social Science, the Chinese University of Hong Kong, Shenzhen 518172, China; 3 Physical Education and Sports Science Academic Group, National Institute of Education, Nanyang Technological University, Singapore 637551, Singapore; cxlilee@gmail.com; 4 Department of Sports Science and Physical Education, The Chinese University of Hong Kong, Hong Kong 999077, China; liyezou123@cuhk.edu.hk; 5 Academy of Martial Arts and Traditional Sports, Beijing Sports University, Beijing 100084, China; 6 Institute on Aging, Seoul National University, Seoul 08826, Korea

**Keywords:** Tai Chi, Qigong, Yoga, cognition, older, research synthesis

## Abstract

*Background*: As the situation of cognitive aging is getting worse, preventing or treating cognitive decline through effective strategies is highly important. This systematic review aims to investigate whether mind-body exercise is an effective approach for treating cognition decline. *Methods*: Searches for the potential studies were performed on the eight electronic databases (MEDLINE, Scopus, Web of Science, SPORTDiscus, CINAHL, PsycArtilces, CNKI, and Wanfang). Randomized controlled trials (RCTs) examining the effect of mind-body exercise on cognitive performance in older adults were included. Data were extracted and effect sizes were pooled with 95% confidence intervals (95% CI) using random-effects models. The Physiotherapy Evidence Database Scale was employed to examine the study quality. *Results*: Nineteen RCTs including 2539 elders (67.3% female) with fair to good study quality were identified. Mind-body exercise, relative to control intervention, showed significant benefits on cognitive performance, global cognition (*Hedges’g* = 0.23), executive functions (*Hedges’g* = 0.25 to 0.65), learning and memory (*Hedges’g* = 0.37 to 0.49), and language (*Hedges’g* = 0.35). In addition, no significant adverse events were reported. *Conclusion*: Mind-body exercise may be a safe and effective intervention for enhancing cognitive function among people aged 60 years or older. Further research evidence is still needed to make a more conclusive statement.

## 1. Introduction

Aging is thought to be the time-based progressive deterioration of physiological functions in organs and tissue that influences human survival and fertility [1]. To date, aging-related health problems (e.g., cardiovascular disease, hypertension, arthritis, and Alzheimer’s disease) have become a serious and global issue, along with the growing of aging and life expectancy. Among these health problems, cognitive aging, which can affect a wide range of cognitive functions such as memory, processing speed, learning, understanding and decision making, is becoming a public health concern [2]. Thus, preventing or treating cognitive decline through effective strategies is highly urgent and critical.

In an effort to address this issue, scholars have focused on preventing or alleviating the rate of cognitive decline using different approaches (e.g., memory training [3], music therapy [4], and exercise therapy [5,6,7]). In the meantime, growing evidence suggests that aerobic exercise is associated with enhanced cognition [8,9,10,11]. Previous studies have shown that aerobic exercise improves the cognitive function at three levels: the systemic level (attention and learning), the molecular level (neurotrophins), and the cellular level (synaptic plasticity) [12]. For instance, research has demonstrated that aerobic exercise could prevent hippocampal volume loss among older adults because of the high level of Brain-Derived Neurotrophic Factor (BDNF) [13]. Another study found that aerobic exercise has a positive effect on cognition for people with mild cognitive impairment (MCI) through elevating the neurotrophic factors levels and decreasing the concentration of inflammatory parameters [14].

Similar to other types of physical exercise, mind-body exercise (i.e., Tai Chi, Qigong, Yoga, and Pilates) involves a variety of actions such as stretching and relaxation of skeletal muscles, as well as coordinated body and regular breathing movements. Additionally, meditative states are also involved in mind-body exercise to regulate attention and consciousness [15]. Recently, mind-body exercise has attracted scholars’ attention because of its effectiveness in treating diseases and secondary conditions such as mental illness, mood disorder, balance problems and ill-being [16,17,18,19]. However, no review study has investigated the impact of mind-body exercise on cognitive performance, and only few reviews have been independently carried out that have focused on the efficacy of Tai Chi, Yoga or Pilates on cognitive function in older adults [20,21]. Moreover, as several recent empirical studies have been performed to examine the effect of mind-body exercise on cognitive performance, there is a need to update existing meta-analyses. Finally, despite Tai Chi and Qigong originating in China, previous reviews did not include articles in the Chinese language. Therefore, this systematic review aims to examine the effects of mind-body exercise on cognitive performance among the elderly. It is hoped that the findings of this review will provide practitioners with evidence for the design of programs for the prevention of cognitive decline, as well as identifying some gaps for future research.

## 2. Materials and Methods

### 2.1. Search Strategy

Searches for the potential studies were performed on the eight electronic databases (MEDLINE, Scopus, Web of Science, SPORTDiscus, CINAHL, PsycArticles, CNKI, and Wanfang) from inception until 29 August, 2018. Three groups of search terms, including “elderly” OR “older people” OR “older adults” OR “aging” OR “senior” AND “cognitive function” OR “cognition” OR “recall” OR “processing” OR “awareness” OR “comprehension” OR “attention” OR “memory” OR “verbal fluency” OR “executive function” OR “language” AND “Tai Chi” OR “Taiji” OR “Qigong” OR “Baduanjin” OR “Yoga” OR “Pilates” OR “mind body exercise” were combined for search. Furthermore, hand searches were used to identify the extra studies from published reference lists. This meta-analysis was conducted in accordance with the Preferred Reporting Items for Systematic Reviews and Meta-Analyses (PRISMA) guideline [22].

### 2.2. Study Selection Criteria

The inclusion criteria for eligible studies were as follows: (1) randomized controlled trial (RCT); (2) participants aged 60 or older; (3) the mind-body intervention included Tai Chi, Qigong, Yoga, and/or Pilates; (4) at least one cognitive outcome was reported and corresponding data were available for effect size calculation; and (5) publications in either English or Chinese language. Studies were excluded if: (1) they were non-randomized controlled trails, case reports, published abstracts, conference proceedings or reviews; (2) the intervention group included training components (e.g., medication, health education) that are not parts of mind-body exercise; or (3) cognitive outcomes were not reported.

### 2.3. Data Extraction and Collection

To obtain the eligible studies in this meta-analysis, two reviewers (Y.Z. and C.X.) independently screened the titles, abstracts and full texts according to the predetermined standards. In the same way, two reviewers (YZ and CX) also extracted the following data: author and year, study design, participant characteristics (sample size, age, gender distribution), intervention program (training type, training time, duration of intervention), and outcomes. In the case of discrepancies, a consensus would be reached by consulting a third reviewer (L.Y.).

### 2.4. Study Quality Assessment

The methodology quality of the included studies was independently assessed by two reviewers (YZ and CX) using the Physiotherapy Evidence Database (PEDro) scale [23]. The scale consists of 11 items: eligibility criteria (not scored), random allocation, concealed allocation, groups similar at baseline, blinding of therapist, blinding of assessors, less than 15% dropouts, intention-to-treat analysis, between-group statistical comparisons, and point measures and variability data. Studies were classified as having excellent (9–10), good (6–8), fair (4–5) or poor (<4) quality, respectively.

### 2.5. Statistical Analysis

The Comprehensive Meta-Analysis software was used to perform the meta-analysis. The standardized mean difference or effective size (*Hedges’g*) was computed. Of note, the original study with two control groups led to 2 effect sizes. Effect sizes across individual trials were synthesized for each outcome using the random-effects model with 95% confidence intervals (CI) and *p* value. The magnitudes of effect size were classified as small (0.2–0.49), medium (0.5–0.79), and large (0.8 or more), respectively. *I*^2^ statistic was used to estimate the heterogeneity across studies. The *I*^2^ values were divided into three levels: small (25%), moderate (50%), and high (75%). Additionally, to examine potential variables that may account for the effect of mind-body exercise on cognitive performance, moderator analysis was conducted according to either the categorical (cognitive status) or continuous predictor (total training time). Publication bias was assessed using the Egger’s test and a significant *p* value indicates publication bias.

## 3. Results

### 3.1. Search Results

**Figure 1:**
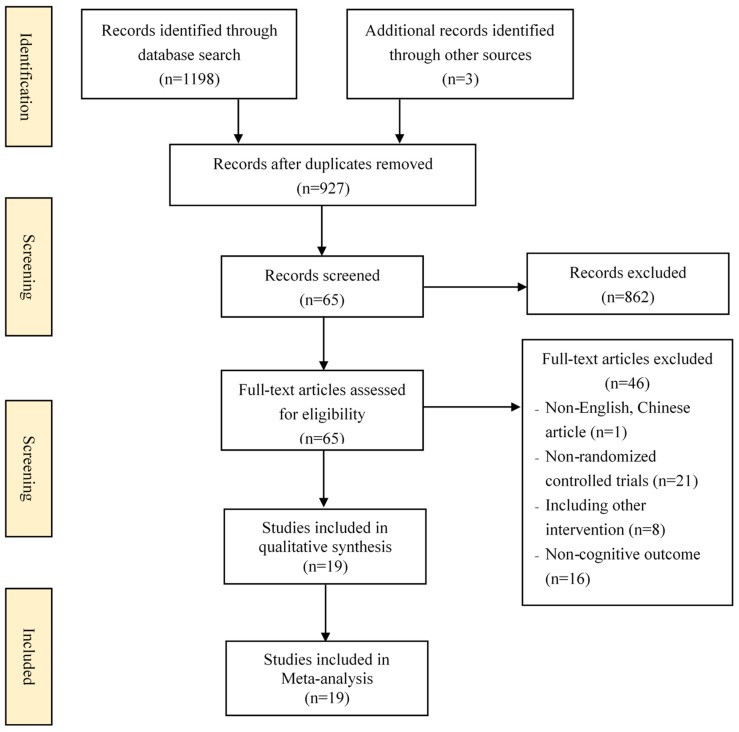
Flow diagram of the study selection process.

Figure 1 describes the process of study selection. Electronic and manual searches returned 1201 records. Duplicates (*n* = 274) were firstly removed. Secondly, 65 potential studies were retained after screening the titles and abstracts. Finally, 46 studies were further excluded after reading through full texts and 19 eligible studies met the inclusion criteria were included in this review.

### 3.2. Study Characteristics

The detailed characteristics of 19 included studies [24,25,26,27,28,29,30,31,32,33,34,35,36,37,38,39,40,41,42] are provided in Table 1. Fifteen studies [25,27,28,29,30,31,32,33,34,35,36,37,38,39,42] had only one control group, and the remaining studies [24,26,40,41] involved two or three control groups. Thus, 24 independent effect sizes were obtained. Across the included studies, a total of 2539 participants (67.3% female) were involved, and the sample size of each study ranged from 28 to 456. The participants’ mean age ranged from 60 to 84 years, and some of them had diseases such as cognitive impairment, stroke and depression. Tai Chi, Yoga, Qigong and Pilates programs were employed in twelve [24,25,27,28,29,33,36,37,39,40,41,42], four [26,31,32,35], two [34,38] and one [30] studies, respectively. The duration of intervention ranged from 8 to 52 weeks. Meanwhile, varied training session time (20 to 120 min) and weekly training sessions (1 to 7) were reported. The control group included various interventions, such as health education, attention training, and stretching and toning exercise. Only two studies [26,32] used the wait-list control. 

### 3.3. Outcomes Measured

The main indicators of cognitive function evaluation included 5 main domains: global cognition (i.e., Mini-Mental State Examination [MMSE]), executive function (i.e., Trial Making Test [Part A, Part B], Stroop Test, Digit Span [Forward, Backward]), learning and memory (Hopkins Verbal Learning Test [HVLT], Rey’s Auditory Verbal Learning Test [RAVLT], California Verbal Learning Test II [CVLT]), visuospatial ability (Rey’s Complex Figure Test [CFT], Clock-drawing Task, Lowenstein Occupational Therapy Cognitive Assessment [LOTCA-G]) and language (Boston Naming Test [BNT]).

### 3.4. Study Quality Assessment

Table 2 presents the methodology quality of the included studies. The quality of selecting eligible studies ranged from fair to good (score range: 4 to 8 points). In total, 14 of the 19 included studies were classified as good, representing low risk bias [24,25,26,29,32,33,34,36,37,38,39,40,41,42]. Half of the included studies reported the concealed allocation, blinding assessor and more than 85% follow-up of at least one outcome. It was noted that 7 studies [26,27,28,30,31,35,36] did not adopt the intention-to-treat analysis to deal with the missing data.

### 3.5. Mind-Body Exercise and Global Cognitive Function

Ten trials [27,29,33,36,37,38,39,40,42] assessed the effect of mind-body exercise on global cognition with the pooled result (*Hedges’g* = 0.47, 95% CI 0.15 to 0.78, *p* = 0.003, *I*^2^ = 88%). Sensitivity analysis indicated that one trial [39] had a very large effect size (*Hedge’s g* = 4.95) compared with the rest trails *Hedges’g* = 0.03 to 0.63). The pooled result, after excluding the trial [39], revealed that the intervention group had a small improvement in MMSE compared with the control group (*Hedges’g* = 0.23, 95% CI 0.08 to 0.39, *p* = 0.003, *I*^2^ = 49.47%) (Table 3). Egger’s test suggested that there was no publication bias (*p* = 0.10).

### 3.6. Mind-Body Exercise and Executive Function

Eight trials [28,31,32,36,38,41] assessed the effect of TMT-A (Table 3). The result of meta-analysis indicated a moderate improvement in mind-body exercise group compared with the control group (*Hedges’g* = 0.65, 95% CI 0.20 to 1.10, *p* < 0.001, *I*^2^ = 90.00%). In terms of TMT-B (Table 3), the aggregated result from eight trials [31,32,35,36,38,41] suggested that a significant difference was observed between the mind-body exercise and the control intervention (*Hedges’g* = 0.46, 95% CI 0.26 to 0.67, *p* < 0.001, *I*^2^ = 50.90%). According to Egger’s test, no publication bias was observed on TMT-A (*p* = 0.06) but there was significant publication bias on TMT-B (*p* < 0.001).

For digit span outcomes, they were evaluated in two arms (digit span-forward and digit span-backward) (Table 3). Eight trails [24,32,36,41] investigated the effect of mind-body exercise on digit span-forward, and the aggregated result suggested a small effect in favor of the mind-body exercise group (*Hedges’g* = 0.25, 95% CI 0.09 to 0.42, *p* = 0.003, *I*^2^ = 45.07%). Regarding digit span-backward scores reported among eight trials [24,25,30,32,41], a pooled analysis suggested that a small effect favoring the mind-body exercise group (*Hedges’g* = 0.29, 95% CI 0.08 to 0.49, *p* < 0.001 and *I*^2^ = 28.52%). Egger’s test suggested that there was no significant publication bias on digit span-forward (*p* = 0.10) and digit span-backward (*p* = 0.76).

The pooled result from seven trails [26,32,35,41] suggested that a small improvement of Stroop test in the mind-body exercise intervention in comparison to the control group (*Hedegs’g* = 0.32, 95% CI 0.015 to 0.49, *p* < 0.001, *I*^2^ = 0%; Table 3). Egger’s test suggested that there was no publication bias (*p* = 0.88).

### 3.7. Mind-Body Exercise and Language

Eight trials [24,30,35,36,41] measured the language ability for older adults in Table 3. When compared to the control group, a small positive effect as compared with the control was detected after receiving the mind-body intervention (*Hedges’g* = 0.35, 95% CI 0.14 to 0.56, *p* = 0.001, *I*^2^ = 48.30%). Egger’s test suggested that there was no publication bias (*p* = 0.20).

### 3.8. Mind-Body Exercise and Learning and Memory

The immediate recall and delayed recall that are indictors of the learning and memory function were reported in seven and eight trials, respectively (Table 3). In terms of the immediate recall [26,32,35,41], the pooled result demonstrated a small and significant benefit in favor of the mind-body exercise (*Hedges’g* = 0.37, 95% CI 0.20 to 0.54, *p* < 0.001, *I*^2^ = 0%). As for delayed recall, the synthesized result from eight trials [25,29,30,32,35,41] suggested that the mind-body exercise was more effective than the control intervention and the effect was close to moderate (*Hedges’g* = 0.49, 95% CI 0.29 to 0.69, *p* < 0.001, *I*^2^ = 23.15%). Egger’s test suggested that there was no publication bias on both immediate recall (*p* = 0.26) and delayed recall (*p* = 0.91).

### 3.9. Mind-Body Exercise and Visuospatial Ability

Eight trials [24,30,35,36,41] investigated visuospatial ability (Table 3), and the synthesized results indicated a small improvement of visuospatial ability that was significantly related to mind-body exercise (*Hedges’g* = 0.18, 95% CI 0.02 to 0.35, *p* = 0.03, *I*^2^ = 0%). Egger’s test suggested that there was no publication bias (*p* = 0.83).

### 3.10. Moderator Analysis

A sub-group analysis was performed to examine the effects of mind-body exercise on cognitive performance based on the categorical (cognitive status: cognitive impairment including MCI and dementia vs. non-cognitive impairment) and continuous predictor (total training time) in Table 4 and Table 5, respectively. Collectively, there were no statistical group differences in cognitive functions except for language (*Q* = 5.45, df = 1, *p* = 0.02) (Table 4). Moreover, mind-body exercise seemed to be more effective for elderly people without cognitive impairment. In term of continuous predictors, the results of meta-regression revealed that total training time is a significant and positive predictor of global cognition (*β* = 0.0005, *Q* = 4.25, df = 1, *p* = 0.04), executive function (TMT-A [*β* = 0.00007, *Q* = 12.69, df = 1, *p* < 0.01], TMT-B [*β* = 0.00006, *Q* = 9.38, df = 1, *p* < 0.01], digit span-forward [*β* = 0.00006, *Q* = 9.35, df = 1, *p* < 0.01]), and language (*β* = 0.00005, *Q* = 5.01, df = 1, *p* = 0.03).

### 3.11. Adverse Events

Seventeen out of the 19 studies recorded no adverse events, and there were only two adverse events [26,35] (Table 3). One participant was diagnosed with groin muscle strain while practicing Yoga [26]. Another person felt dizziness during Yoga training [35].

## 4. Discussion

Our review summarized the evidence regarding the effects of mind-body exercise on cognitive performance for adults aged 60 years and older. Although different types of mind-body interventions (i.e., Tai Chi, Yoga, Pilates, and Qigong) were used across 19 included studies, all of these exercise programs involved core actions and breathing techniques (e.g., stretching, lying, and abdominal breathing) [15,43,44]. Despite varying exercise programs and participant characteristics (e.g., stroke, MCI, and depression), the findings of this review generally suggested that mind-body exercise could be an effective and safe method in improving older adults’ cognitive performance.

### 4.1. Global Cognition

A small effect of mind-body exercise on global cognitive function was detected in the present review. Worthy of noting is that only two out of the ten included studies demonstrated that mind-body exercise significantly benefited to holistic cognition of healthy elders or older adults with MCI [27,37]. The insignificant findings of individual studies may be attributed to the small number of training sessions (e.g., [29]) and/or the inclusion of other types of exercise (e.g., resistance training) in the control group (e.g., [33,36,40,42]). By comparison, the meta-analytic result of mind-body exercise on global cognition was congruent with prior reviews and meta-analysis examining the efficacy of Pilates, Yoga or Tai Chi on global cognitive function [21,45,46]. For example, in Wayne and colleagues’ meta-analysis, in which they included 4 RCTs (Tai Chi vs. blanket control) and a small treatment effect was observed among elders [21].

### 4.2. Executive Function

The executive functions involve advanced cognitive processes (e.g., planning, attention, working memory, multitasking, and purposive action), which are deemed necessary for behavioral control in humans [47]. In our review, the TMT [Part A and Part B], Stroop test and digit span (forward, backward) were used to assess executive function [24,25,26,28,30,31,32,35,36,38,41], and mild to moderate improvements were obtained. This result provides the very first piece of synthesized evidence that mind-body exercise may enhance executive function in senior people. When participating in mind-body exercise, trainees are required to remember the new skills and movement patterns, and to recall the various movements and their sequences. The frontal lobe, as part of the brain areas, may thus regulate the executive function to complete tasks through mind-body exercise [48,49]. Moreover, it is highly possible that there is a reciprocal effect between mind-body movement and executive function [47].

### 4.3. Other Functions

In a normal aging process, there is a decline of language function such as verbal fluency, verbal retrieval and naming tasks [50]. We found that the positive effect of mind-body exercise on language functions was stronger among healthy participants than counterparts with cognitive impairments. The moderation effect is tentative, given only a small number of trials were involved in the analysis. On the other hand, the detected effect may be due to the fact that language functions tend to remain in a better condition among people have no cognitive impairments than those who have cognitive impairments during the aging process [51]. This finding suggests that it would be difficult to reverse or maintain older adults’ language function once they are diagnosed with cognitive impairments.

For learning and memory, our findings were consistent with previous clinical trials [29,32,35,41], showing that mind-body exercise significantly improved the performance in immediate and delayed recall. Early research has shown that healthy older volunteers who received Tai Chi and Baduanjin training had greater changes in hippocampus and improvement in memory performance than elderly who did not receive any intervention [52,53]. Given there is a significant relationship between hippocampal volume and memory function [13,54], participants may increase their hippocampus volume after practicing mind-body exercise.

Likewise, the synthesized result indicated that mind-body exercise significantly improved participants’ visuospatial ability, which is crucial for independent activities in life. Previous studies have demonstrated that aerobic exercise improved the visuospatial ability through enhancing the functional connectivity [55]. Given mind-body exercise is considered to be one kind of aerobic exercise, it might have a similar effect in enhancing functional connectivity among elderly.

### 4.4. Possible Mechanisms

Some plausible hypotheses may be used for explaining the positive effects of mind-body exercise on cognitive functions among elderly. Mind-body exercise emphasizes the coordination of body movements and rhythmic breathing, attention of feeling, weight shifting as well as the connection between the body and mind [15,25,31]. The characteristics of mind-body exercise have been found to be associated with increased hippocampus volumes and frontal lobes stimulation [53,56]. As such, a body of studies supports the hypothesis that hippocampus and frontal lobes may play a critical role in preserving cognitive function [49,52,53]. Additionally, some evidence demonstrates that physical activity contributes to elevate the levels of plasma biomarkers such as BDNF and tumor necrosis factor-α, which are important for cognitive function enhancement [13,25]. Indeed, practicing mind-body exercise involves skill learning processes such as memorizing skills or imitating others [57,58]. Lots of beneficial effects were gained from this kind of skill-related learning activity [59,60]. For example, an increase in brain regions (e.g., the cingulate cortex, insula, corpus callosum, and sensorimotor cortex) was observed after learning how to dance [60]. Therefore, all this evidence provided us with a better understanding of the mechanistic and molecular basis of how mind-body exercise improves cognitive function.

### 4.5. Limitations

Our review is subject to several limitations. First, several significant heterogeneities were observed in several of the cognitive outcomes. The heterogeneities may be attributed to the use of various assessment tools while evaluating the same cognitive outcome. In addition, the varied participant characteristics may also contribute to heterogeneity. Second, the current review was limited to cognitive outcomes. Other indexes, such as stress and emotion, that are closely associated with cognition among older adults were not evaluated in this review [2]. Finally, different programs were included as a comparison to mind-body exercise interventions, making the interpretation of meta-analytic results difficult.

## 5. Conclusions

A relatively large number of studies with fair to good methodological quality have been conducted to evaluate the efficacy of mind-body exercise on cognitive functions among elderly. The findings from this review generally suggest that mind-body exercise could be safe and effective in enhancing cognitive function for people aged 60 years or older. More RCTs with good to excellent research quality assessing different types of cognitive functions are needed to provide additional evidence. In addition, both a short-term and follow-up evaluation should be included in future research to evaluate the lasting effect of mind-body exercise on cognitive outcomes.

## Figures and Tables

**Table 1:** Characteristics of randomized controlled trials in the meta-analysis.

Study	Participants			Intervention Protocol		Duration	Outcomes (Instrument)	Safety
	Healthy Status	Sample Size (female%)	Age (years)	Experiment	Control			Adverse Effect
Taylor-Piliae et al. (2010) [24]	Healthy	132 (54.5%)	69	5 × 45 min/week, TC	C1: 5 × 30–55 min/week, Vigorous walking (30 min), resistance (light hand weights and elastic strap) and flexibility training (10–25 min) C2: 1 × 90 min/week, Attention-control	24 weeks	Language (BNT) Executive function: (WAIS)	No
Sungkarat et al. (2018) [25]	MCI	66 (86.4%)	67.9	3 × 50 min/week, TC	1 × 60 min/week, Health education about cognitive impairment and fall prevention	24 weeks	Learning and memory (WMS) Visuospatial ability (BDT) Executive function (WAIS) Executive function (TMT)	No
Oken et aL (2006) [26]	Healthy	135 (74.8%)	72	1 × 90 min/week structured Yoga class + daily home Yoga practice	C1: 5 × 60 min/week, Walking (moderate intensity) C2: Wait-list	24 weeks	Executive function (SCWT) Learning and memory (10-word list learning task)	1 person with groin muscle strain
Sun et al. (2015) [27]	Healthy	138 (75.4%)	69	2 × 60 min/week, TC	Playing cards or singing	24 weeks	Global cognition (MMSE)	No
Nguyen et al. (2012) [28]	Healthy	96 (50%)	68.9	2 × 60 min/week, TC	Routine daily activities	24 weeks	Executive function (TMT)	No report
Lavretsky et al. (2013) [29]	Major depression	73 (61.6%)	70.5	1 × 120 min/week, TC	1 × 120 min/week, Health education on depression, stress, sleep, and health-related issues	10 weeks	Global cognition (MMSE) Learning and memory (CVLT) Executive function (SCWT)	No
Greblo Jurakic et al. (2017) [30]	MCI	28 (100%)	70.4	3 × 60 min/week, Pilates	3 × 30 min/week, HUBER^®^ training	8 weeks	Attention (MoCA) Learning and memory (MoCA) Language (MoCA) Visuospatial ability (MoCA)	No
Gothe et al. (2017) [31]	Healthy	118 (48.3%)	62	3 × 60 min/week, Yoga	3 × 60 min/week, Stretching and muscle strength, 10–12 repetitions for each exercise (i.e., resistance bands, bicep curls, tricep extensions, and flutter kicks)	8 weeks	Executive function (TMT)	No
Sivakumar et al. (2015) [32]	Stroke and Psychosis	120 (60%)	75	3–4 × 60 min/week, Yoga	Wait-list	24 weeks	Executive function (*WMS)* Executive function (SCWT) Executive function (TMT) Visuospatial ability (CFT) Learning and memory (RAVLT) Learning and memory (COWA)	No
Hwang et al. (2016) [33]	Falling	456 (66.7%)	73	1 × 60 min/week, TC	1 × 60 min/week, Stretching, muscle strengthening (i.e., hip extensors, abductors/knee flexors and extensors, and ankle dorsiflexors and plantar flexors), and balance training at increasing difficulty levels	24 weeks	Global cognition (MMSE)	No
Tsang et al. (2013) [34]	Frailty	116 (75%)	84	2–3 × 60 min/week Qigong + daily home Qigong practice	Newspaper reading	12 weeks	Attention (LOTCA)	No
Eyre et al. (2017) [35]	MCI	79 (65.8%)	68	1 × 60 min/week, Yoga	Memory training	12 weeks	Learning and memory (WMS) Visuospatial ability (Rey-O) Executive function (TMT) Language (BNT)	1 side effect (dizziness)
Lam et al. (2011) [36]	MCI	389 (76.3%)	78	3–7 × >30 min/week, TC	3–7 × >30 min/week, Stretching exercise	8 weeks	Global cognition (MMSE) Executive function (CTMT) Language (CVFT) Executive function (WMS)	No
Siu et al. (2018) [37]	MCI	160 (73.6%)	≥60	2 × 60 min/week, TC	Usual care (i.e., recreational activities, general physical mobility, and social activities)	16 weeks	Global cognition (MMSE)	No
Cai et al. (2018) [38]	MCI	58	67	5 × 90 min/week, Qigong	Usual care (i.e., recreational activities, general physical mobility, and social activities)	24 weeks	Global cognition (MMSE) Executive function (CTMT) Executive function (WMS)	No
Zhou et al. (2016) [39]	Dementia	40 (55%)	67	5 × 60 min/week, TC	Jogging (HR≈120/min)	32 weeks	Global cognition (MMSE)	No
Dechamps et al. 2010 [40]	Dementia	160 (78.1%)	82	4 × 30 min/week, TC	C1: 2 × 30–40 min/week, Mild intensity exercises (e.g., cycling and knee elevations, arm rising, and circle drawing) C2: usual care (i.e., no restriction in medical care, physical activity, physiotherapy, or any health care support)	24 weeks	Global cognition (MMSE)	No
Mortimer et al. 2012 [41]	Healthy	120 (66.7%)	68	3 × 50 min/week, TC	C1: 3 × 50 min/week, aerobic exercise (brisk walking) C2: 3 × 60 min/week, social interaction (lively discussion with each other) C3: no intervention	40 weeks	Executive function (SCWT) Learning and memory (CAVLT) Language (BNT) Executive function (TMT) Visuospatial ability (CDT) Executive function (WAIS)	No
Tsai et al. 2013 [42]	MCI & Osteoarthritic Knee	55 (72.7%)	79	4 × 20–40 min/week, TC	Health education and activities (e.g., sharing travel experiences, hobbies, and collections)	20 weeks	Global cognition (MMSE)	No

Note: AE = adverse effect; TC = Tai Chi; C = Control group; MCI = Mild Cognitive Impairment; HR = Heart rate; BDT = Block Design Test; BNT = Boston Naming Test; WAIS = Wechsler Adult Intelligence Scale; TMT = Trail Making Test; WMS = Wechsler Memory Scale; SCWT = Stroop Color and Word Test; CVLT = California Verbal Learning Test; MoCA = Montreal Cognitive Assessment; CFT = Complex Figure Test; RAVLT = Rey’s Auditory Verbal Learning Test; COWA = Controlled Oral Word Association Test; LOTCA = Lowenstein Occupational Therapy Cognitive Assessment; Rey-O = Rey Osterrieth test; CDT = Clock drawing test; CVFT = Category Verbal Fluency Tests; CAVLT = Chinese Auditory Verbal Learning Test.

**Table 2:** Methodological quality of the included studies (PEDro analysis).

Study	Score	Methodological Quality	PEDro Item Number										
			1	2	3	4	5	6	7	8	9	10	11
Taylor-Piliae et al. 2017 [24]	7	Good	✓	✓	✓	✓			✓		✓	✓	✓
Sungkarat et al. 2018 [25]	8	Good	✓	✓	✓	✓			✓	✓	✓	✓	✓
Oken et al. 2006 [26]	7	Good	✓	✓	✓	✓			✓	✓		✓	✓
Sun et al. 2015 [27]	5	Fair	✓	✓		✓				✓		✓	✓
Nguyen et al. 2012 [28]	4	Fair	✓	✓		✓						✓	✓
Lavretsky et al. 2012 [29]	8	Good	✓	✓	✓	✓			✓	✓	✓	✓	✓
Greblo Jurakic et al. 2017 [30]	5	Fair	✓	✓		✓				✓		✓	✓
Gothe et al. 2017 [31]	5	Fair	✓	✓		✓				✓		✓	✓
Sivakumar et al. 2013 [32]	6	Good	✓	✓	✓	✓					✓	✓	✓
Hwang et al. 2016 [33]	7	Good	✓	✓	✓	✓			✓		✓	✓	✓
Tsang et al. 2013 [34]	7	Good	✓	✓		✓			✓	✓	✓	✓	✓
Eyre et al. 2017 [35]	5	Fair	✓	✓		✓			✓			✓	✓
Lam et al. 2011 [36]	6	Good	✓	✓		✓			✓	✓		✓	✓
Siu et al. 2018 [37]	6	Good	✓	✓		✓				✓	✓	✓	✓
Cai et al. 2018 [38]	6	Good	✓	✓		✓				✓	✓	✓	✓
Zhou et al. 2016 [39]	6	Good	✓	✓		✓				✓	✓	✓	✓
Dechamps et al. 2010 [40]	7	Good	✓	✓	✓	✓				✓	✓	✓	✓
Mortimer et al. 2012 [41]	6	Good	✓	✓		✓				✓	✓	✓	✓
Tsai et al. 2013 [42]	7	Good	✓	✓		✓			✓	✓	✓	✓	✓

Note: Studies were classified as having excellent (9–10), good (6–8), fair (4–5) or poor (<4) quality. Scale of item score: ✓, present. The PEDro scale criteria are (1) eligibility criteria; (2) random allocation; (3) concealed allocation; (4) similarity at baseline on key measures; (5) participant blinding; (6) instructor blinding; (7) assessor blinding; (8) more than 85% retention rate of at least one outcome; (9) intention-to-treat analysis; (10) between-group statistical comparison for at least one outcome; and (11) point estimates and measures of variability provided for at least one outcome.

**Table 3:** Synthesized results for the effects of mind-body exercise vs control intervention.

Outcomes		Number of Trials	Meta-Analysis			Heterogeneity			Publication Bias
			*Hedges’g*	95% CI	*p*-Value	*I*^2^ %	*Q*-Value	df(*Q*)	Egger’s Test (*p*)
Global cognition	MMSE	9	0.23	0.08 to 0.39	0.003	49.47%	15.83	8	0.10
Executive function	TMT-A	8	0.65	0.20 to 1.10	0.005	90.00%	70.06	7	0.06
	TMT-B	8	0.46	0.26 to 0.67	0.000	50.90%	14.25	7	0.00
	DS-Forward	9	0.25	0.09 to 0.42	0.003	45.07%	14.57	8	0.01
	DS-Backward	8	0.29	0.08 to 0.49	0.006	28.52%	9.79	7	0.76
	Stroop Test	7	0.32	0.15 to 0.49	0.000	0%	5.99	6	0.88
Learning and memory	Immediate recall	7	0.32	0.15 to 0.49	0.000	0%	6.18	6	0.39
	Delayed recall	8	0.49	0.29 to 0.69	0.000	23.95%	9.20	7	0.89
Language	Naming test	8	0.35	0.14 to 0.56	0.001	48.30%	13.54	7	0.20
Visuospatial ability		8	0.18	0.02 to 0.35	0.030	0%	1.82	7	0.83

TMT = Trial Making Test; DS = Digit Span.

**Table 4:** Moderator analysis for the effects of mind-body exercise vs control intervention (categorical predictor).

Outcomes		Population	Number of Trials	Sub-Analysis			between-Group Homogeneity		
				*Hedges’g*	95% CI	*I*^2^ %	*q*-Value	df(*Q*)	*p*-Value
Global cognition	MMSE	MCI	3	0.28	−0.05 to 0.61	69.06%	0.09	1	0.77
		Non-MCI	6	0.22	0.03 to 0.42	46.62%			
Executive function	TMT-A	MCI	2	0.35	−0.30 to 0.97	81.56%	0.84	1	0.36
		Non-MCI	6	0.75	0.16 to 1.35	90.18%			
	TMT-B	MCI	3	0.42	0.01 to 0.84	71.35%	0.11	1	0.74
		Non-MCI	5	0.50	0.30 to 0.71	4.06%			
	DS-Forward	MCI	3	0.19	−0.07 to 0.46	66.45%	0.47	1	0.49
		Non-MCI	6	0.31	0.10 to 0.52	19.78%			
	DS-Backward	MCI	2	0.19	−0.21 to 0.58	0%	0.26	1	0.61
		Non-MCI	6	0.31	0.05 to 0.56	46.21%			
	Stroop Test	MCI	1	0.37	−0.07 to 0.81	0%	0.06	1	0.81
		Non-MCI	6	0.31	0.11 to 0.51	15.67%			
Learning and memory	Immediate recall	MCI	2	0.44	−0.24 to 1.11	79.43%	0.23	1	0.63
		Non-MCI	5	0.27	0.06 to 0.47	0%			
	Delayed recall	MCI	3	0.66	0.22 to 1.11	51.33%	1.01	1	0.31
		Non-MCI	5	0.41	0.20 to 0.62	0%			
Language	Naming test	MCI	4	0.14	−0.02 to 0.30	0%	5.45	1	0.02
		Non-MCI	5	0.49	0.24 to 0.74	26.36%			
Visuospatial ability		MCI	3	0.27	−0.03 to 0.56	0%	0.50	1	0.48
		Non-MCI	5	0.14	−0.06 to 0.34	0%			

TMT = Trial Making Test; DS = Digit Span; MCI = Mild Cognitive Impairment; MMSE = Mini-Mental State Examination.

**Table 5:** Moderator analysis for the effects of mind-body exercise vs control intervention (continuous predictor).

Outcomes		Continuous Predictors	Number of Trials	β	95% CI	*Q*-Value	df(*Q*)	*p*-Value
Global cognition	MMSE	Total training time	9	0.00005	0.00000 to 0.00011	4.25	1	0.039
Executive function	TMT-A	Total training time	8	0.00007	0.00003 to 0.00011	12.69	1	0.000
	TMT-B	Total training time	8	0.00006	0.00002 to 0.00010	9.38	1	0.002
	DS-Forward	Total training time	9	0.00006	0.00002 to 0.00009	9.35	1	0.002
	DS-Backward	Total training time	8	−0.00004	−0.00015 to 0.00007	0.48	1	0.489
	Stroop Test	Total training time	7	0.00002	−0.00004 to 0.00009	0.47	1	0.492
Learning and memory	Immediate recall	Total training time	7	0.00002	−0.00005 to 0.00008	0.24	1	0.622
	Delayed recall	Total training time	7	−0.00002	−0.00009 to 0.00004	0.46	1	0.496
Language	Naming test	Total training time	8	0.00005	0.00001 to 0.00010	5.01	1	0.025
Visuospatial ability		Total training time	8	−0.00002	−0.00008 to 0.00005	0.24	1	0.625

TMT = Trial Making Test; DS = Digit Span; MMSE = Mini-Mental State Examination.
